# Sex-Determined Alteration of Frontal Electroencephalographic (EEG) Activity in Social Presence

**DOI:** 10.3390/life13020585

**Published:** 2023-02-20

**Authors:** Anna Soiné, Peter Walla

**Affiliations:** 1 CanBeLab, Psychology Department, Webster Vienna Private University, Praterstrasse 23, 1020 Vienna, Austria; 2 Medical Neurosciences, Charité Universitätsmedizin Berlin, Charitéplatz 1, 10117 Berlin, Germany; 3 Faculty of Psychology, Freud CanBeLab, Sigmund Freud University, Sigmund Freud Platz 1, 1020 Vienna, Austria; 4 Faculty of Medicine, Sigmund Freud University, Sigmund Freud Platz 3, 1020 Vienna, Austria; 5 School of Psychology, Newcastle University, University Drive, Callaghan, NSW 2308, Australia

**Keywords:** electroencephalography, EEG, social presence, anterior cingulate cortex, ACC, sex-determined activation, sex differences, female, male, women, men, sex, gender, social neuroscience

## Abstract

This study represents a follow-up event-related potential (ERP) analysis of a prior investigation. The previous results showed that participants had most negative-tending ERPs in the mid-frontal brain region during exposure to neutral emotion pictures (compared to negative and positive pictures) while being accompanied by a significant other person (social presence condition). The present analysis aimed at investigating potential sex differences related to this phenomenon. Female and male participants’ brain activity data from the previous study were analyzed separately for one representative mid-frontal electrode location selected on the basis of having the highest significance level. As a result, only female participants showed significantly more negative-tending potentials in response to neutral pictures, compared to both other emotion categories (positive and negative) in the social presence condition. This was not found in male participants. The respective ERP effect was most dominant at 838 ms post stimulus onset, which is slightly later than the effect found in the prior study. However, this result is interpreted as evidence that the general effect from the prior study can be understood as a largely female phenomenon. In line with the prior study, the present results are interpreted as a predominantly female activation in the mid-frontal brain region in response to neutral picture stimuli while being accompanied by a significant other person (social presence condition). Although only speculative, this would align with previous studies demonstrating sex-related hormonal and structural differences in the anterior cingulate cortex (ACC). In general, ACC activation has been associated with an integrative weighting function in ambiguous social settings, which makes sense given the ambiguous nature of neutral pictures in combination with a social presence condition.

## 1. Introduction

Although the existence of sex-related differences in the brain appears to have reached scientific consensus, many neural dissimilarities remain controversial [1,2]. This is highly obvious with the conflicting nature of theories on sex-dependent brain activation in emotion-related tasks, particularly. Sex-dependent brain activation differences in emotion-related processing are generally indicated to be complex and region-specific, and their neural circuits and resulting affective and cognitive implications have not yet been fully identified [3]. In the following, a selection of neurophysiological findings including distinct neuroanatomical structures are highlighted. While processing social affective stimuli, males showed greater activation in the right medial frontal gyrus, the left fusiform gyrus, the right parahippocampal gyrus, and the amygdala [4]. Females exhibited heightened neural activity in the right subcallosal gyrus. Similar studies found males to display heightened activity in the inferior frontal gyrus, the right paracingulate gyrus, the right insula, and the left thalamus, as well as again in the right medial frontal gyrus, the fusiform gyrus, and the amygdala [5,6,7]. Furthermore, males demonstrated greater left posterior cingulate and right inferior temporal gyrus activity while viewing positive social videos. Negative social videos elicited higher activation in the left middle temporal region in males compared to females. In response to positive affective pictures, males demonstrated higher right hemispheric activity in the ACC, the medial frontal gyrus, the superior frontal gyrus, and the superior temporal gyrus than females. No such differences were found while processing negative affective pictures [8]. The majority of all those prior studies used functional Magnet Resonance Imaging (fMRI) to measure brain activation. A meta-analysis found that across such brain imaging studies, sex differences were mainly reported in relation to positive and/or negative emotion stimuli [9], but not to neutral pictures. Interestingly, the authors of this meta-analysis indeed reported only effects found as a result of utilizing fMRI or positron emission tomography (PET). Only a few other studies on sex differences have used actual neurophysiological methods such as electroencephalography (EEG) and magnetencephalography (MEG) (e.g., [10,11]), which are both well known for their excellent temporal resolution. Given the relatively high speed of information processing in the brain, functional differences between females and males might be short (fractions of a second), which makes those methods particularly interesting. For instance, brain amplitude differences have been reported about females showing significantly higher N1 (negativity at around 100 ms post stimulus) and N2 (negativity at around 200 ms post stimulus) amplitudes compared to males when exposed to negative stimuli [12]. Further EEG studies found a significantly longer latency and higher amplitude of the P450 ERP component [13], as well as a heightened N2 ERP component with a greater activity of the fusiform face area (FFA) and the extrastriate body areas (EBA) to reflect sex differences [14]. The respective results were interpreted as reflecting the higher attention of female participants towards social stimuli, which may reflect a female privilege in the automatic processing of social stimuli in principle [15]. This is supported by enhanced female brain activation in response to social affective stimuli in general [16,17,18].

This lack of consistency in current findings on sex-related brain functioning, as depicted above, reveals the necessity for more naturalistic experiments, which should target specific aspects of sex differences in brain activation, especially in relation to social constructs. Naturalistic experiments thereby refer to experimental conditions, which include stimuli and situations of ‘natural’ human life as accurately as possible. As human brain structures have evolutionarily evolved to detect and react to social, economic, and survival-related stimuli, it may be assumed that cues not associated with such essential decisions require less attention. Consequently, insignificant situations may elicit less brain activity. Most of the experiments underlying the conflicting findings on neurophysiological sex differences in emotion-related processing made use of virtual affective and social stimuli [3,4,5,6,7,8,9,10]. The insignificance of such cues for individual survival-related choices likely influenced the ambiguous nature of the results. This is because un-naturalistic experimental conditions (i.e., virtual affective stimuli) solely represent tiny fractions of naturalistic situations (i.e., complex social situation) and may therefore only partially recruit the relevant neural circuits.

Naturalistic experimental variables, in contrast, seem to have the potential to recruit relevant brain mechanisms more accurately [19,20]. Acute stress, as an example of a naturalistic stimulus, impacts emotion-related perception for males and females in the opposite direction. Women displayed enhanced functional connectivity in the amygdala and between the insula and the FFA for both, the control, and the stress condition. The broader brain circuitry involved was only revealed through the inclusion of naturalistic independent variable, namely acute stress. Crucially, ERP findings of this preceding study [20] revealed that the mid-frontal brain region (most likely ACC-related) shows greater activity levels during exposure to neutral pictures when participants were accompanied by a significant other person (i.e., a social presence condition), which can be understood as a highly relevant real-life and thus naturalistic social condition. The authors concluded that their social presence condition altered the processing of neutral pictures, because of their ambiguous nature, which led participants to evaluate neutral stimuli more carefully as mistakes made in front of others are more costly than when made alone [20]. However, this study did not aim to investigate any potential sex-differences, which now became the main focus of the present re-analysis of the data from the prior study.

The combination of neutral pictures being processed differently during actual social presence with the notion that naturalistic stimuli such as actual social presence might specifically lead to sex-dependent brain activity differences formed the basis for the present study. Thus, the present study aimed at investigating whether social presence alters cortical activation in response to neutral picture processing differently for females and males.

## 2. Materials and Methods

### 2.1. Participants

The data analysis underlying this research is based on 30 data sets collected for a prior study [20]. For the original study, respective EEG data sets were collected together with skin conductance response (SCR) data and self-report data. Due to this multi-methods approach, artifacts in one of the three measurements led to the exclusion of 11 data sets resulting in 19 remaining data sets (11 = female, 8 = male) in the study by Soiné et al. [20]. This would have been too few data to perform a gender comparison. In the present analysis, we excluded SCR and self-report data, which led to a higher number of data sets, making it possible to compare female and male participants.

The present analysis is now based on data that were collected from 27 right-handed, healthy, English-speaking participants in the frame of the prior study (females = 14, males = 13) [20]. All participants confirmed not to be restricted by current health issues or mind-altering states (no psychopathologies or mind-altering drugs) and to have normal or corrected to normal vision. For the social presence condition all participants were asked to bring a same sex significant other person with them. The study was permitted by the ethics committee of the Webster University in Saint Louis, MI, USA.

To determine the intimacy of each pair of participants, the “Inclusion of the Other in the Self” (IOS) Scale was used [21]. Consisting of a 7-point Likert scale (with 7 representing the highest intimacy), all pairs of participants ranking each other as 5 or higher were included in the study.

### 2.2. Stimuli

The stimulus material was described in detail in the prior study [20]. In summary, pre-rated pleasant, unpleasant and neutral pictures were presented on a computer monitor, while the participants’ brain potential changes were recorded. The presented pictures were taken from the OASIS database. They were pre-rated on a 7-point Likert-scale (i.e., 1 = very unpleasant to 7 = very pleasant for valence; 1 = not arousing at all to 7 = very arousing for arousal) [22,23]. According to those pre-ratings, two on average equally rated picture sets were established for the two respective conditions (i.e., alone versus social presence condition). Ninety pictures per condition (i.e., 30 pictures per emotion category—pleasant, neutral, and unpleasant) were used for the experiment. The experiment was designed with the E-Prime 2.0^®^ software package (Psychology Software Tools311 23rd Street Ext., Suite 200, Sharpsburg, PA 15215-2821, USA). For more details about the statistically accurate division of the pictures for the different categories and conditions please refer to Soiné et al. 2021 [20].

During the experiment, the participants viewed all stimuli on a Dell E2214hb 21.5 inch widescreen LED LCD monitor with black background. One trial included the presentation of a blank black screen (1 s), a fixation cross (1 s), a blank black screen (1 s), the corresponding picture chosen by the software for the trial (1 s), a blank black screen (1 s), and, finally, a valence and an arousal rating screen. To show all 90 pictures per condition, 90 trials were conducted. 

### 2.3. Data Collection

To measure cortical activity, the Geodesic EEGTM System 400 with a silver chloride HydroGel Geodesic Sensor Net of 64 electrodes was applied to the participant’s head (see [20]). The EGI Net Amps 400 amplifier with a built-in Intel chip continually sampled all potential changes at a rate of 1000 Hz. An offline bandpass filter from DC to 30 Hz was applied. The Net Station 5.4 software was used to obtain the data. More details, including the whole procedure, can be found in Soiné et al. [20].

Baseline brain activity was measured during the second blank black screen and the ERP measurement, in response to the pre-rated pictures, followed during the third blank black screen. The purpose of the fixation cross was to reduce ocular artifacts by allowing the participants time to blink. Please refer to the prior study by Soiné et al. [20] for more details.

### 2.4. Data Analysis

EEGDISPLAY 6.4.9 (by Ross Fulham) using an offline bandpass filter from 0.1 to 30 Hz was used to process and clean EEG data. Baseline-corrected epochs were generated, and the relevant data from 100 ms prior to the inception of every stimulus to 1000 ms after the stimulus inception were extracted. Visible artifacts were removed manually and all amplitudes denoted as beyond 75 mV by the software were removed by an automated threshold detection algorithm. Grand averages were computed according to the 2 × 3 experimental design (2 social conditions (alone and with social presence) and 3 picture emotion categories (pleasant, unpleasant and neutral). All averages were re-referenced to the common average. The 27 valid data sets were grouped into female (*N* = 14) and male (*N* = 13) participants. All neurophysiological data were then down-sampled, resulting in 25 data points, each data point averaged across 40 ms for each condition and for each electrode.

First, an analysis including regions of interest was done. For this purpose, seven regions were defined and within each region several electrodes were pooled together. The regions were defined according to anatomical areas as follows: “left-frontal” (9 averaged electrodes: 10,11,12,13,14,17,18,19,64 (numbers are taken from the EGI System)), mid-frontal” (4 averaged electrodes: 3, 6, 8, 9), “right-frontal” (9 averaged electrodes: 1, 2, 5, 56, 57, 58, 59, 60, 61), “mid-central” (6 averaged electrodes: 4, 7, 16, 21, 41, 51), “left-parietal” (7 averaged electrodes: 24, 25, 26, 27, 28, 29, 30), “mid-parietal” (7 averaged electrodes: 33, 34, 35, 36, 37, 38, 39) and “right-parietal” (7 averaged electrodes: 42, 44, 45, 46, 47, 48, 52). The aim of this was to test if a potential sex difference was region-specific and to find out which time points demonstrated a significant sex difference for both social conditions (alone and social presence). A repeated-measures ANOVA, including all regions of interest as a within-subject factor with 7 levels, emotion condition as a within-subject factor with 3 levels and sex as a between-subject factor with 2 levels was calculated for each of the 25 data points. The respective calculation revealed three time points for which significant region*emotion*sex interactions occurred. Those were 638 ms, 838 ms und 878 ms after stimulus onset, with the highest significance level for 838 ms (*p* = 0.028). Consequently, further analysis was focused on this time point only. Because the present paper aimed at testing if the finding from the prior study by Soiné et al. [20] varies as a function of sex, further analysis was done only for the mid-frontal region, where visual inspection of the newly generated ERPs resulted in clear evidence that females contributed dominantly to the previously described effect [20].

The final and most relevant analysis was based on one of the two electrode positions from the prior study [20] that were found as showing highest significance levels with respect to the described neurophysiological effect. For the selected mid-frontal electrode (electrode 8) amplitude means related to all three emotion categories for both sexes and for both social conditions were compared separately by calculating paired-sample *t*-tests. Due to multiple comparisons, Bonferroni correction was applied to adjust the initial α of 0.05. Given that three comparisons were calculated within each sex group and per social presence condition, the adjusted α resulted in 0.016. See respective results in Table 1 and Table 2.

## 3. Results

### EEG Results

**Figure 1:**
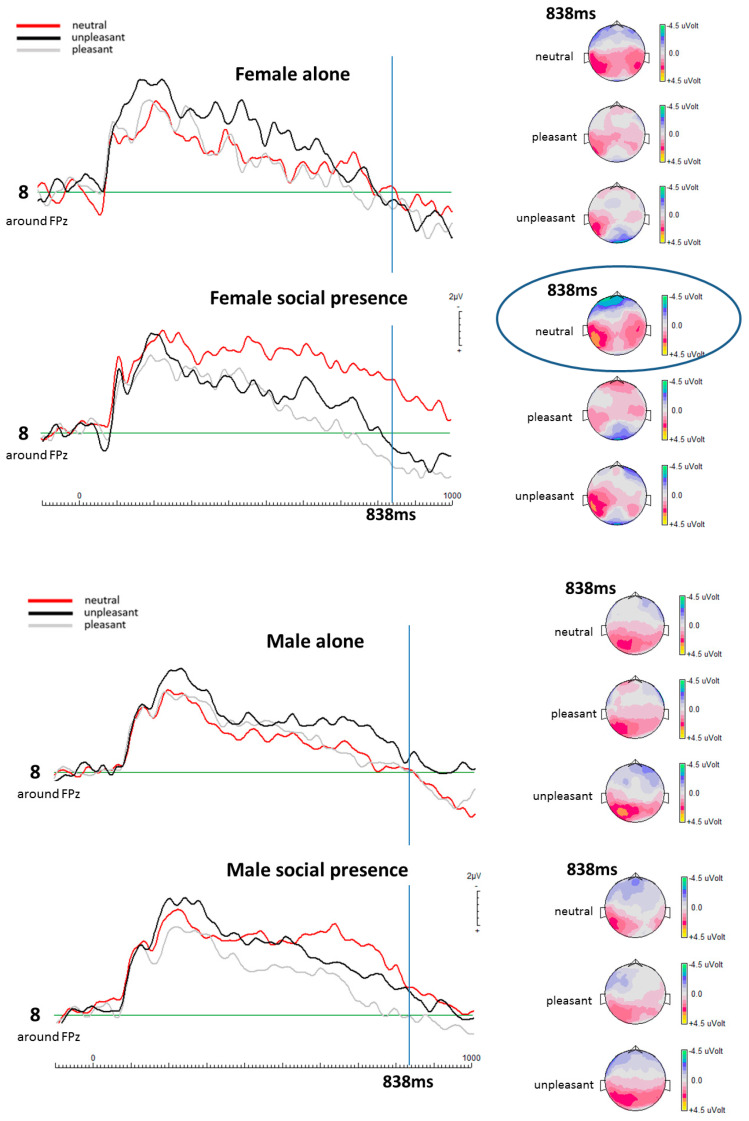
Left: ERPs calculated from brain potentials recorded at electrode location 8 (mid-frontal location) for all three emotion categories and both experimental conditions are shown for females and males separately. Right: Respective topographical maps for each valence category and both experimental conditions are shown for females and males. For females but not for males, the ERPs and their topographic display show clearly that neutral pictures in the social presence condition elicit the most negative brain potentials at mid- and left-frontal electrode locations.

Paired sample *t*-tests, contrasting the ERP results between the differently valenced pictures (pleasant, neutral, unpleasant) for the social presence condition and for the alone condition showed significant differences that follow a certain pattern with respect to sex (see Figure 1 and Table 1). Contrasting the ERPs of the female and male participants in the social presence condition revealed significantly more negative going potentials at the selected mid-frontal electrode for the female participants in response to the neutral pictures (see Figure 1).

At 838 ms after stimulus onset, ERPs of female participants in the social presence condition showed significant differences for the neutral and pleasant picture conditions with a *p*-value of 0.013 for mid-frontal electrode 8. Similarly, a significant difference was found between the neutral and unpleasant picture conditions for female participants in the social presence condition with a *p*-value of 0.013. No significant differences were detected between the unpleasant and pleasant picture conditions for female participants in the social presence condition (electrode 8: *p* = 0.492). In contrast, male participants showed no significant differences in cortical activity between any picture category pairs in the social presence condition at the same electrode location. Consequently, female participants exhibited a significantly heightened cortical activity in the mid-frontal region in response to neutral pictures during the social presence condition, whereas male participants did not show any such effect (see Table 1).

At 838 ms in the alone condition, *t*-test results showed no significant difference for the female participants between the neutral and pleasant picture conditions, with a *p*-value of 0.634 for mid-frontal electrode 8. Similarly, no significant difference was detected between the neutral and unpleasant picture conditions with a *p*-value of 0.977, as well as for the unpleasant and pleasant picture conditions with *p* = 0.320. Male participants also did not show any significant differences for any picture category pairs (see Table 2).

## 4. Discussion

### 4.1. Social Presence, Ambiguous Stimuli, and the Present Results 

Brain activation-related sex differences in response to affective stimuli have become a greater focus of research within the last decades. However, most research has focused solely on investigating sex-reliant brain activation mechanisms associated with inanimate emotional stimuli, such as photos and videos [4,5,6,7,8,9,10,11,12]. Even though those studies provide important insights into affective brain functioning, only naturalistic stimuli resembling significant social, economic, or survival-related choices may have the potential to fully recruit relevant brain mechanisms prone to sex differences [19,20]. As recently reported, social presence seems to represent such a naturalistic independent variable providing significant cues for human life. In this earlier study [20], it was found that social presence elicited heightened mid-frontal cortical activity during exposure to neutral stimuli in a group of male and female participants. LORETA analysis within this earlier study attributed the observed increased activation to the ACC. This finding is supported by various brain imagining studies including EEG studies suggesting a multifunctional key role of the ACC in gauging significant social stimuli [24,25,26,27,28,29,30]. This is of importance for an individual’s beneficial adjustment to internal and environmental circumstances such as the individual’s social inclusion status. Consequently, ambiguous stimuli (i.e., neutral pictures) in social presence are indicated to imply decisive cues for social decisions [20]. The current study re-analyzed data from this prior study [20] to test whether sex differences can be found. The re-evaluation now indicates that social presence most dominantly alters brain responses to ambiguous emotion-related stimuli (i.e., neutral pictures) in female participants. This is reflected in significantly enhanced cortical activity in the mid-frontal cortical region in female participants compared to their male counterparts, while exposed to neutral pictures in the social presence condition. This neurophysiological effect was neither found for female participants in the alone condition, nor for male participants in the alone and the social presence condition. It is, therefore, concluded that the effect reported in Soiné et al. [20] largely emerged from female participants. It is to be noted that the present female-dominant effect appears approximately 130ms later than the effect found across both sexes. Despite this temporal difference in acitvation, we continue to suggest the ACC as strongly contributing to the heightened activation in the social presence condition during exposure of neutral pictures. This argument is supported by numerous neuroimaging studies, which have associated mid-frontal ERP-effects with changes in ACC activation [24,25,31,32,33,34,35,36,37].

### 4.2. Sex Differences in the Area of the ACC at Cellular, Structural, and Network Level

Sex differences in the ACC and associated structures have been documented at cellular, structural, and network levels. Female reproductive hormones have been shown to impact cellular function, thereby structurally and functionally modulating neural circuitry [38]. Controlling for the hormonal phase of women (i.e., menopause, menstrual cycle, and ovarian steroid manipulations), fMRI as well as PET studies have related two female hormones, estradiol and progesterone, to have neuroregulatory effects on various structures. These associated areas, including the ACC, play a role in the processing of affective states and (social) situation-specific decision making, as employed in the present study. Therefore, these regions are considerably impacted by the neuroregulatory effects of female hormones [38]. Estradiol and progesterone are indicated to have the most potent effect on the dorsolateral prefrontal cortex (dlPFC) and the hippocampus (working memory) [39,40,41], the orbitofrontal cortex (OFC) and the amygdala (reward system) [42,43,44,45], the insula (salience system) [46], the medial prefrontal cortex (mPFC) and the rostral ACC (rACC) (default mode) [47,48,49,50], as well as on the rest of the ACC, the amygdala, and the OFC (emotional processing) [51,52,53,54,55,56].

On a structural level, the hippocampus, parietal and occipital regions, as well as the locus coeruleus and areas of the cingulate cortex differ in males and females with the latter accounting for a heightened receipt and processing of information within the female limbic system [2,57]. Females show increased grey matter volume within the cingulate cortex [58], specifically within the cingulate sulcus, whereas males show a greater volume in the paracingulate sulcus [59]. Significantly greater peak density was demonstrated in the female subcallosal anterior cingulate [3], while reduced right ACC volume in boys (but not girls) was linked to aggressive and defiant behavior [60]. On the network level, sex differences have been documented in brain circuits responsible for affect-related cognitive control as well as stress. While cognitive emotional control elicited diminished amygdala activation in men, women display heightened activation in the ACC, the ventral striatum and the frontal regions [61,62]. When viewing negative emotional cues, females were shown to display increased activation in the area of the cingulate cortex indicating a female advantage in adjusting to negative events [9,63]. Furthermore, perceived stress causes heightened PFC and reduced OFC activation for men, whereas women demonstrate increased activation of limbic structures, including the ACC [64].

### 4.3. Interpretation of the Present Results

In light of the prior study [20], the present results, although still speculation-based, indicate increased ACC activation in female participants compared to their male counterparts when exposed to neutral pictures while accompanied by a significant other person (social presence condition). It is suggested that the increased neural activity associated with neutral pictures relates to the ACC’s role in processing several components of uncertainty and ambiguity [65,66]. This heightened ACC activation may reflect an individuals’ personal ambiguity attitudes in the choice between alternative strategies with the intention of choosing the best value option [26,65]. The potential of social presence to alter ACC activation is linked to the fact that mistakes made in the presence of others are more costly than made when alone. Hence, social situations may catalyze the pressure for individuals to find the best suitable option [67,68]. This specific activation of the ACC in ambiguous social settings as indicated in the present results aligns with previous findings. These findings attribute an integrative weighting function accountable for controlling adaptive social cognition mechanisms as well as mediating the value of social stimuli to the ACC [24,25,26,27,28,29,66,69,70]. This includes a personal cost-benefit evaluation and an estimate of the motivation of others [67,71]. Neural responses to social affective stimuli and resulting situation-specific decisions are largely generated within the ACC [30,67,71]. These processes are controlled through various neuroregulatory effects including female reproductive hormones, sex-dependent differences in the volume of brain structures and differences in brain circuitry (i.e., for the execution of emotion-related cognitive control) [3,47,48,49,50,51,52,53,54,56,58,59,61,62].

Evolution has long shaped affective, cognitive, and behavioral processes differently for men and women. This, for instance, is shown in the tend-and-befriend response (versus fight-or-flight response), which is more dominantly seen in females compared to males. While tending is referred to as self-protecting and safety promoting nurturant activities, befriending involves social networking in support of the former. Female reproductive hormones, endogenous opioid peptide mechanisms and oxytocin have potent neuroregulatory effects on brain structures including the ACC, which in turn underlie the tend-and-befriend response [47,49,72]. These different sex-related adaptive mechanisms can be observed in animal herds, where females conjointly tend, while men compete for the status of the alpha male. While in the animal kingdom such behaviors still have evolutionary advantages, human survival does no longer depend on such adaptive evolutionary mechanisms, thanks to the safety of civilization. However, a female tendency of tending-and-befriending versus a male tendency of fight-and-flight can still be seen. For example, when asked to describe advantages of workplace friendships in times of stress, women focused on social and emotional support, while men emphasized benefits for their productiveness and prospective career [73]. Given the fact that those sex-dependent behavioral tendencies are still evident in modern society, the question arises, whether this behavior can be purely subject to evolutionary mechanisms?

Besides evolution, sociocultural norms are a powerful force determining emotional expressions as well as behavior by defining what is inappropriate and what is desirable. Sociocultural norms further influence how we perceive and value ourselves and others, how we interpret situations and act on them and how we are perceived by others [74]. Due to their impact on such personal perception patterns, norms have the potential to alter affections, cognitions, and behavior. A change in affective, cognitive, and behavioral aspects is accompanied by an alteration in brain activity, which again underlies neuroregulatory parameters on cellular levels. Historically, the norms enforced by society have differentially influenced the affections, cognitions, and behavior of men and women. Stereotypical viewpoints, which were enforced by society over centuries include women being perceived as less competitive, warmer, and more tending versus men being more aggressive, competitive, and less emotional [75]. Given their significant impact on human life, sociocultural norms may well play an important role in shaping sex-related neural differences on cellular, structural, and network levels.

With regar to the current study, the heightened ACC activity found in female participants in the social presence condition when viewing neutral pictures can be interpreted as reflecting affective cortical activity patterns driven by social adaptive mechanisms. As humans are social animals, social adaptation has always been essential. This is reflected in the structure of the human brain, which evolved for optimal situation-specific decision-making to ensure social benefits (for an overview of social decision-making mechanisms in the ACC see Soiné et al., 2021 [20]). While evolution framed the major social brain mechanism for survival, sociocultural norms coined social rules in civilization. Therefore, the impact of sociocultural norms on behavior, and therefore on cortical affect regulation and cognition, is most potent in social presence.

## 5. Conclusions

The present study found a maximal increase in mid-frontal cortical activity in female participants at 838 ms after stimulus onset when exposed to neutral pictures while being accompanied by a significant other person (social presence condition). This aligns with previous studies suggesting sex-related hormonal and structural brain differences. We attribute the present differences between females and males in social presence mainly to evolutionary and sociocultural aspects, which enforced variations in female and male affective, cognitive, and behavioral patterns over time. In an age shaped by the redefinition of traditional gender identities and roles it is important to first understand how sex-related differences in female and male affect, cognition, and behavior emerged. Acknowledging the various biological differences between the two opposite sexes on the spectrum will hopefully give rise to a less categorical perception of the sexes. This includes the recognition of the continuous nature of hormonal levels, molecular structures, degrees of XY chromosome expression, and anatomical givens. Further research is necessary to foster a more holistic understanding of the biological basis underlying non-traditional and not binary gender identities and roles. 

Beyond this, health, disease and treatment success can be determined by a variety of interlinked cellular, structural, and functional signaling cascades related to sex. As the Western medical system is currently tailored to the average male body and its symptomology, it does not do justice to the individual medical needs of different sexes on the spectrum. This affects many individuals daily, because gender medicine has not yet advanced enough for a gender-fair adjustment of diagnostic criteria and drug dosages. Hence, taking a more gender equitable approach towards Western medicine may be one decisive factor influencing treatment success rates in the present and future. For instance, entangling the biological basis underlying the spectrum of sexes and its physiological correlates may represent a key for the development of more sustainable treatment options. This may significantly reduce the societal burden of repeated health care costs due to mistreatment. The present results contribute one piece to this puzzle by revealing how female and male brains exhibit differential magnitudes of cortical potentials in response to neutral pictures in a social presence condition. 

## Figures and Tables

**Table 1:** *p*-values of ERPs for female and male participants in the social presence condition at electrode 8 at 838ms post stimulus onset between the respective valence categories. Significant values are marked bold and in red color.

	Social Presence Condition	
**Female Participants**	**at 838 ms**	
	**Valence Categories**	***p*** **-Value**
Electrode 8 (mid-frontal)	**Neutral–Pleasant**	***p* = 0.013 (t(13) = −0.429)**
	**Neutral–Unpleasant**	***p* = 0.013 (t(13) = 0.466)**
	Unpleasant–Pleasant	*p* = 0.492 (t(13) = 0.631)
**Male Participants**	**at 838 ms**	
	**Valence Categories**	***p*** **-Value**
Electrode 8 (mid-frontal)	Neutral–Pleasant	*p* = 0.547 (t(12) = 0.594)
	Neutral–Unpleasant	*p* = 0.787 (t(12) = 0.447)
	Unpleasant–Pleasant	*p* = 0.701 (t(12) = 0.497)

**Table 2:** *p*-values of ERPs for female and male participants in the alone condition at mid-frontal electrode 8 at 838 ms post stimulus onset between the respective valence categories. There are no significant values.

	Alone Condition	
**Female Participants**	**at 838 ms**	
	**Valence Categories**	***p*** **-Value**
Electrode 8 (around FPz)	Neutral–Pleasant	*p* = 0.634 (t(13) = 0.537)
	Neutral–Unpleasant	*p* = 0.977 (t(13) = 0.347)
	Unpleasant–Pleasant	*p* = 0.320 (t(13) = 0.754)
**Male Participants**	**at 838 ms**	
	**Valence Categories**	***p*** **-Value**
Electrode 8 (around FPz)	Neutral–Pleasant	*p* = 0.735 (t(12) = 0.722)
	Neutral–Unpleasant	*p* = 0.415 (t(12) = 0.995)
	Unpleasant–Pleasant	*p* = 0.510 (t(12) = 0.775)

## Data Availability

No new data were created or analyzed in this study. Data sharing is not applicable to this article.

## References

[1] Kret M.E., De Gelder B. (2012). A review on sex differences in processing emotional signals. Neuropsychologia.

[2] Raz N., Gunning-Dixon F., Head D., Rodrigue K.M., Williamson A., Acker J.D. (2004). Aging, sexual dimorphism, and hemispheric asymmetry of the cerebral cortex: Replicability of regional differences in volume. Neurobiol. Aging.

[3] Wagner T.D., Phan K.L., Liberzon I., Taylor S.F. (2003). Valence, gender, and lateralization of functional brain anatomy in emotion: A meta-analysis of findings from neuroimaging. Neuroimage.

[4] Fusar-Poli P., Placentino A., Carletti F., Landi P., Allen P., Surguladze S., Benedetti F., Abbamonte M., Gasparotti R., Barale F. (2009). Functional atlas of emotional faces processing: A voxel-based meta-analysis of 105 functional magnetic resonance imaging studies. J. Psychiatry Neurosci..

[5] Lee T.M., Liu H.L., Chan C.C., Fang S.Y., Gao J.H. (2005). Neural activities associated with emotion recognition observed in men and women. Mol. Psychiatry.

[6] Rahko J., Paakki J.J., Starck T., Nikkinen J., Remes J., Hurtig T., Kuusikko-Gauffin S., Mattila M.-L., Jussila K., Jansson-Verkasalo E. (2010). Functional mapping of dynamic happy and fearful facial expression processing in adolescents. Brain Imaging Behav..

[7] Wrase J., Klein S., Gruesser S.M., Hermann D., Flor H., Mann K., Braus D.F., Heinz A. (2003). Gender differences in the processing of standardized emotional visual stimuli in humans: A functional magnetic resonance imaging study. Neurosci. Lett..

[8] Fine J.G., Semrud-Clikeman M., Zhu D.C. (2009). Gender differences in BOLD activation to face photographs and video vignettes. Behav. Brain Res..

[9] Stevens J.S., Hamann S. (2012). Sex differences in brain activation to emotional stimuli: A meta-analysis of neuroimaging studies. Neuropsychologia.

[10] Duregger C., Bauer H., Cunnington R., Lindinger G., Deecke L., Lang W., Walla P., Dirnberger G. (2007). EEG evidence of gender differences in a motor related CNV study. J. Neural. Transm..

[11] Walla P., Hufnagl B., Lindinger G., Deecke L. (2001). Physiological evidence of gender differences in word recognition: A magnetoencephalographic (MEG) study. Cogn. Brain Res..

[12] Gardener E.K.T., Carr A.R., MacGregor A., Felmingham K.L. (2020). Sex differences and emotion regulation: An event-related potential study. PLoS ONE.

[13] Orozco S., Ehlers C.L. (1998). Gender differences in electrophysiological responses to facial stimuli. Biol. Psychiatry.

[14] Proverbio A.M., Zani A., Adorni R. (2008). Neural markers of greater female responsiveness to social stimuli. BMC Neurosci..

[15] Proverbio A.M. (2017). Sex differences in social cognition: The case of face processing. J. Neurosci. Res..

[16] Klein S., Smolka M.N., Wrase J., Gruesser S.M., Mann K., Braus D.F., Heinz A., Gruesser S.M. (2003). The influence of gender and emotional valence of visual cues on fMRI activation in humans. Pharmacopsychiatry.

[17] Gard M.G., Kring A.M. (2007). Sex differences in the time course of emotion. Emotion.

[18] Proverbio A.M., Adorni R., Zani A., Trestianu L. (2009). Sex differences in the brain response to affective scenes with or without humans. Neuropsychologia.

[19] Mather M., Lighthall N.R., Nga L., Gorlick M.A. (2010). Sex differences in how stress affects brain activity during face viewing. Neuroreport.

[20] Soiné A., Flöck A.N., Walla P. (2021). Electroencephalography (Eeg) reveals increased frontal activity in social presence. Brain Sci..

[21] Gächter S., Starmer C., Tufano F. (2015). Measuring the closeness of relationships: A comprehensive evaluation of the “inclusion of the other in the self” scale. PLoS ONE.

[22] Kurdi B., Lozano S., Banaji M.R. (2016). OASIS_database_2016. https://www.dropbox.com/sh/4qaoqs77c9e5muh/AABBw07ozE__2Y0LVQHVL-8ca?dl=0.

[23] Kurdi B., Lozano S., Banaji M.R. (2017). Introducing the open affective standardized image set (OASIS). Behav. Res. Methods.

[24] Simmons A., Stein M.B., Matthews S.C., Feinstein J.S., Paulus M.P. (2006). Affective ambiguity for a group recruits ventromedial prefrontal cortex. Neuroimage.

[25] Carter C.S., Braver T.S., Barch D.M., Botvinick M.M., Noll D., Cohen J.D. (1998). Anterior cingulate cortex, error detection, and the online monitoring of performance. Science.

[26] Kolling N., Behrens T.E.J., Wittmann M.K., Rushworth M.F.S. (2016). Multiple signals in anterior cingulate cortex. Curr. Opin. Neurobiol..

[27] Botvinick M., Nystrom L.E., Fissell K., Carter C.S., Cohen J.D. (1999). Conflict monitoring versus selection for-action in anterior cingulate cortex. Nature.

[28] Fritz J., Dreisbach G. (2013). Conflicts as aversive signals: Conflict priming increases negative judgments for neutral stimuli. Cogn. Affect. Behav. Neurosci..

[29] Holroyd C.B., Yeung N. (2013). An Integrative Theory of Anterior Cingulate Cortex Function: Option Selection in Hierarchical Reinforcement Learning. Neural Basis Motiv. Cogn. Control.

[30] Dalgleish T., Walsh N.D., Mobbs D., Schweizer S., Van Harmelen A.L., Dunn B., Dunn V., Goodyer I., Stretton J. (2017). Social pain and social gain in the adolescent brain: A common neural circuitry underlying both positive and negative social evaluation. Sci. Rep..

[31] Li P., Peng W., Li H., Holroyd C.B. (2018). Electrophysiological measures reveal the role of anterior cingulate cortex in learning from unreliable feedback. Cogn. Affect. Behav. Neurosci..

[32] Van Veen V., Carter C.S. (2002). The anterior cingulate as a conflict monitor: FMRI and ERP studies. Physiol. Behav..

[33] Bush G., Luu P., Posner M.I. (2000). Cognitive and Emotional Influence in Anterior Cingulate Cortex. Trends Cogn. Sci..

[34] Lavin C., Melis C., Mikulan E., Gelormini C., Huepe D., Ibañez A. (2013). The anterior cingulate cortex: An integrative hub for human socially-driven interactions. Front. Neurosci..

[35] Eisenberger N.I., Lieberman M.D., Williams K.D. (2003). Does rejection hurt? An fMRI study of social exclusion. Science.

[36] Onoda K., Okamoto Y., Nakashima K., Nittono H., Ura M., Yamawaki S. (2009). Decreased ventral anterior cingulate cortex activity is associated with reduced social pain during emotional support. Soc. Neurosci..

[37] Aarts E., Roelofs A., Van Turennout M. (2008). Anticipatory activity in anterior cingulate cortex can be independent of conflict and error likelihood. J. Neurosci..

[38] Rubinow D.R., Schmidt P.J. (2019). Sex differences and the neurobiology of affective disorders. Neuropsychopharmacology.

[39] Shanmugan S., Loughead J., Cao W., Sammel M.D., Satterthwaite T.D., Ruparel K., Epperson C.N., Gur R.C. (2017). Impact of trypotophan depletion on executive system function during menopause is moderated by childhood adversity. Neuropsychopharmacology.

[40] Berent-Spillson A., Persad C.C., Love T., Tkaczyk A., Wang H., Reame N.K., Smith Y.R., Zubieta J.-K., Frey K.A. (2010). Early menopaus hormone use influences brain regions used for visual working memory. Menopause.

[41] Berman K.F., Schmidt P.J., Rubinow D.R., Danaceau M.A., Van Horn J.D., Esposito G., Weinberger D.R., Ostrem J.L. (1997). Modulation of cognition-specific cortical acivity by gondadal steroids: A position-emission tomotgraphy study in women. Proc. Natl. Acad. Sci. USA.

[42] Macoveanu J., Henningsson S., Pinborg A., Jensen P., Knudsen G.M., Frokjaer V.G., Siebner H.R. (2016). Sex-steroid hormone manipulation reduces brain response to reward. Neuropsychopharmacology.

[43] Bayer J., Bandurski P., Sommer T. (2013). Differential modulatio of acitivity related to the anticipation of monetary gains and losses across the menstrual cycle. Eur. J. Neurosicence.

[44] Dreher J., Schmidt P.J., Kohn P., Furman D., Rubinow D.R., Berman K.F. (2007). Menstrual cycle phase modulates reward-related neural function in women. Proc. Natl. Acad. Sci. USA.

[45] Rupp H.A., James T.W., Ketterson E.D., Sengelaub D.R., Janssen E., Heiman J.R. (2009). Neural activation in women in response to masculinized male faces: Mediation by hormones and psychosexual factors. Evol. Hum. Behav..

[46] Joffe H., Deckersbach T., Lin N.U., Makris N., Skaar T.C., Rauch S.L., Dougherty D.D., Hall J.E. (2012). Metabolic activity in the insular cortex and hypothalamus predicts hot flashes: An FDG-PET study. J. Clin. Endocrinol. Metab..

[47] Arelin K., Mueller K., Barth C., Rekkas P.V., Kratzsch J., Burmann I., Sacher J., Villringer A. (2015). Progesterone mediates brain functional connectivity changes during the menstrucal cycle-a pilot resting state MRI study. Front. Neurosci..

[48] Petersen N., Kilpatrick L.A., Goharzad A., Cahill L. (2014). Oral contraceptives pill use and menstrual cycle phase are associated with altered resting state functional connectivity. Neuroim.

[49] Syan S.K., Minuzzi L., Costesu D., Smith M., Allega O.R., Coote M., Hall G.B., Frey B.N. (2017). Influence of endogenous estradiol, progesterone, allopregnanolone, and dehydroepiandrosterone sulfate on brain resting state functional connectivity across the menstrual cycle. Fertil. Steril..

[50] Hjelmervik H., Hausmann M., Osnes B., Westerhausen R., Specht K. (2014). Resting states are resting traits-an FMRI study on sex differences and menstrual cycle effects in resting state cognitive control networks. PLoS ONE.

[51] Andreano J.M., Touroutoglou A., Dickerson B., Barrett L.F. (2018). Hormonal Cycles, Brain Network Connectivity, and Windows of Vulnerability to Affective Disorder. Trends Neurosci..

[52] Goldstein J.M., Jerram M., Poldrack R., Ahern T., Kennedy D.N., Seidman L.J., Makris N. (2005). Hormonal cycle modulates arousal circuitry in women using functional magnetic resonance imaging. Handb. Clin. Neurol..

[53] Henningsson S., Madsen K.H., Pinborg A., Heede M., Knudsen G.M., Siebner H.R., Al E. (2015). Role of emotional processing in depressive responses to sex-hormone manipulation: A pharmacological fMRI study. Transl. Psychiatry.

[54] Protopopescu X., Hong P., Margaret A., Oliver T., Margaret P., Bruce M., David S., Emily S., Raichle E.M. (2005). Orbitofrontal Cortex Activity Related to Emotional Processing Changes across the Menstrual Cycle. Proc. Natl. Acad. Sci. USA.

[55] Shafir T., Love T., Berent-Spillson A., Persad C., Wang H., Reame N., Frey K., Zubieta J., Smith Y. (2012). Postmenopausal hormone use impact on emotion processing circuitry. Behav. Brain Res..

[56] Toffoletto S., Lanzenberger R., Gingnell M., Sundstrom-Poromaa I., Comasco E. (2014). Emotional and cognitive functional imaging of estrogen and progesterone effects in the female human brain: A systematic review. Psychoneuroendocrinology.

[57] Valentino R.J., Reyes B., Van Bockstaele E., Bangassser D. (2011). Molecular and cellular sex differences at the intersection of stress and arousal. Neuropharmacology.

[58] Good C.D., Johnsrude I., Ashburner J., Henson R.N., Friston K.J., Frackowiak R.S. (2001). Cerebral asymmetry and the effects of sex and handedness on brainstructure: A voxel-based morphometric analysis of 465 normal adult human brains. Neuroimage.

[59] Paus T., Otaky N., Caramanos Z., MacDonalds D., Zijdenbos A., Davirro D., Gutmans D., Holmes C., Tomaiuolo F., Evans A.C. (1996). In vivo morphometry of intrasulcal gray matter in human cingulate, paracingulate, and superior-rostral sulci: Hemispheric asymmetries, gender differences and probability maps. J. Comp. Neurol..

[60] Boes A.D., Tranel D., Anderson S.W., Nopoulos P. (2008). Right anterior-cingulate: A neuroanatomical correlate of aggression and definance in boys. Behav. Neurosci..

[61] Kateri M., Kevin N.O., Iris B.M., John J.D.G., James J.G. (2008). Gender differences in emotion regulation: An fMRI study of cognitive reappraisal. Gr. Process. Intergr. Relations.

[62] Kogler L., Gur R.C., Derntl B. (2015). Sex differences in cognitive regulation of psychosocial achievement stress: Brain and behavior. Hum. Brain Mapp..

[63] Spalek K., Fastenrath M., Ackermann S., Auschra B., Coynel D., Frey J., Gschwind L., Hartmann F., Van der Maarel N., Papassotiropoulos A. (2015). Sex-dependent dissociation between emotional appraisal and memory: A large-scale behavioral and fMRI study. J. Neurosci..

[64] Wang J., Korczykowski M., Rao H., Fan Y., Pluta J., Gur R.C., McEwen B.S., Detre J.A. (2007). Gender difference in neural response to psychological stress. Soc. Cogn. Affect. Neurosci..

[65] Levy I. (2017). Neuroanatomical Substrates for Risk Behavior. Neuroscientist.

[66] Botvinick M.M. (2007). Conflict monitoring and decision making: Reconciling two perspectives on anterior cingulate function. Cogn. Affect. Behav. Neurosci..

[67] Apps M.A.J., Rushworth M.F.S., Chang S.W.C. (2016). The Anterior Cingulate Gyrus and Social Cognition: Tracking the Motivation of Others. Neuron.

[68] Demolliens M., Isbaine F., Takerkart S., Huguet P., Boussaoud D. (2017). Social and asocial prefrontal cortex neurons: A new look at social facilitation and the social brain. Soc. Cogn. Affect. Neurosci..

[69] Rudebeck P.H., Buckley M.J., Walton M.E., Rushworth M.F.S. (2005). Reports 4.

[70] Ridderinkhof K.R., Ullsperger M., Crone E.A., Nieuwenhuis S. (2004). The role of the medial frontal cortex in cognitive control. Science.

[71] Shenhav A., Botvinick M.M., Cohen J.D. (2013). The expected value of control: An integrative theory of anterior cingulate cortex function. Neuron.

[72] Taylor S.E., Klein L.C., Lewi B.P., Gruenewald T.L., Gurung R.A., Updegraff J.A. (2000). Biobehavioral responses to stress in females: Tend-and-befriend, not fight-or-flight. Psychol. Rev..

[73] Morrison R.L. (2009). Are Women Tending and Befriending in the Workplace? Gender Differences in the Relationship Between Workplace Friendships and Organizational Outcomes. Sex Roles.

[74] Wood W., Eagly A.H. (2015). Two Traditions of Research on Gender Identity. Sex Roles.

[75] Clow K.A., Ricciardelli R. (2011). Women and Men in Conflicting Social Roles: Implications from Social Psychological Research. Soc. Issues Policy Rev..

